# Digital coding metamaterials with multi-modulation schemes and beam steering for intra-chip millimeter-wave connectivity

**DOI:** 10.1038/s41598-025-33590-7

**Published:** 2026-01-22

**Authors:** Zhicheng Shen, Sajjad Taravati, Jize Yan

**Affiliations:** School of Electronics and Computer Science, University of Southampton, SO17 1BJ Southampton, United Kingdom

**Keywords:** Electrical and electronic engineering, Electronics, photonics and device physics

## Abstract

In modern wireless communication systems, data transmission is achieved through the collaboration of digital modulation circuits and antennas. Digital baseband signals are first modulated in terms of amplitude, frequency, and phase of the carrier wave and then transmitted directionally via antennas. However, in intra-chip environments, the performance of on-chip antennas is fundamentally constrained by micro-fabrication and integration requirements. As a result, these antennas often exhibit low gain and efficiency and are susceptible to interference among closely spaced transmission channels. To address these limitations, we propose a digital coding metamaterial for direct signal modulation within intra-chip wireless channels, providing an alternative solution to achieve directional signal delivery without depending on the antenna’s intrinsic radiation pattern. The proposed metamateiral can directly convert digital control inputs into discrete phase shifts of a 70 GHz TE-mode surface wave, enabling post-radiation modulation as the electromagnetic wave propagates through the metamaterial. When combined with a single broadcast antenna, multiple metamaterial units can perform simultaneous, multi-directional modulation and transmissions. The proposed metamaterial supports various phase shift modulation schemes, including BPSK, QPSK, and 8-PSK. Furthermore, it enables hybrid modulation and beam steering modes, offering a beam steering range of up to $\pm 28^{\circ }$. The proposed metamateiral presents an innovative method for information routing in intra-chip transmission, helps reduce signal crosstalk under parallel transmission, and expands wireless channel capacity and spectral efficiency.

## Introduction

Intra- and inter-chip wireless interconnects are dedicated to high-speed wireless interconnection between chips to solve the wiring complexity problem. Traditional wired interconnects face limitations such as signal loss, increased energy consumption, and bandwidth constraints as the size and density of transistors increase in advanced integrated circuits (ICs). To address these challenges, wireless intra-chip millimetre-wave connectivity, which involves using wireless technologies for data transmission between components within a chip, has emerged as a promising solution^1–3^.

In early research on intra-chip wireless interconnects, several pronounced limitations were identified under the dense on-chip environment. The high relative permittivity ($\varepsilon _r=11.9$) and low resistivity (10 to 20 $\Omega$.cm) of the silicon substrate resulted in poor radiation efficiency and high transmission loss. Additionally, a radiated signal could propagate through multiple paths, including space radiation, surface waves, and reflected waves, which often result in significant interference and signal dispersion^1,4^. Subsequent research has therefore focused on employing surface-wave propagation as the dominant transmission in intra-chip channels. Where the EM wave radiated from the on-chip antenna is propagating and spreading on the 2D plane along the silicon substrate, which offers more energy-efficient and controllable radiation than the rapid spherical spreading of free-space radiation^1,5^. This can not only enhance energy efficiency but also mitigate multi-path and reflected-wave interference associated with the space-wave propagation path.

**Fig. 1:**
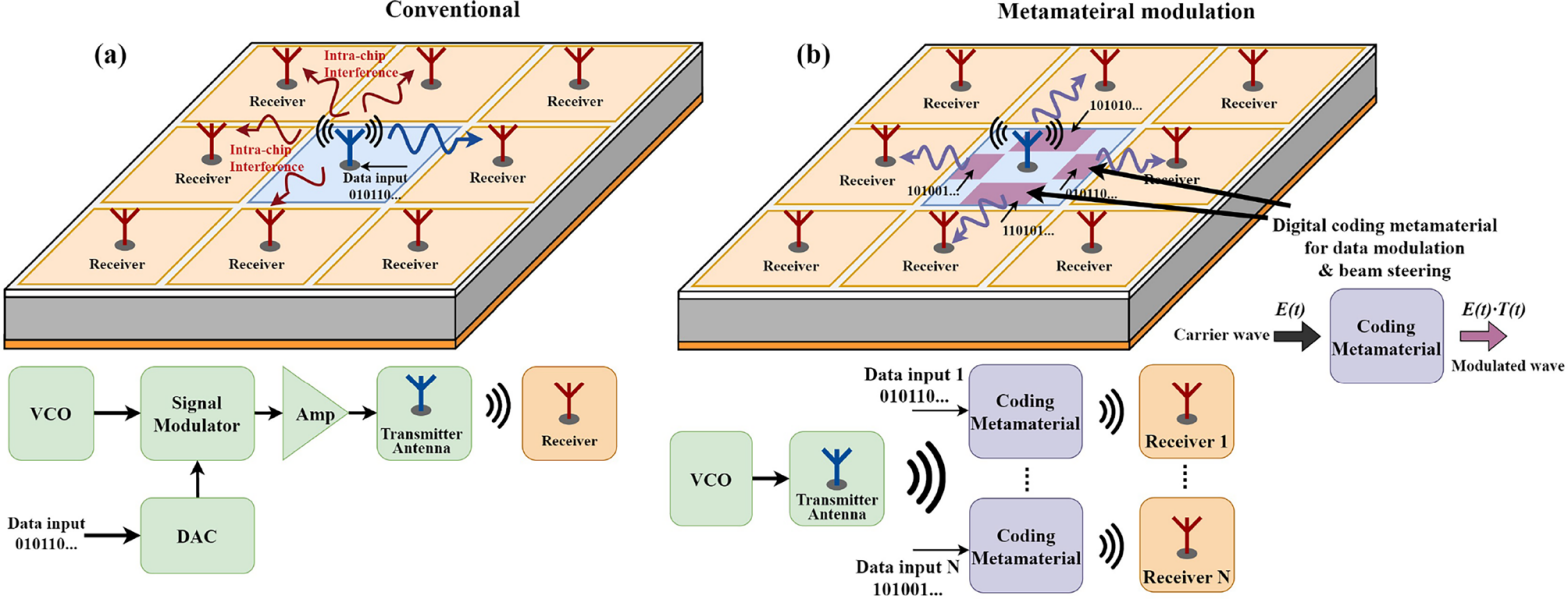
Schematic diagram compares different intra-chip millimetre-wave interconnects based on circuit and metamaterial modulation. (**a**) Conventional wireless interconnect where digital modulation is performed by circuit components before the transmitter antenna. Each TX antenna can only serve one receiver, and low energy efficiency and strong interference to other regions of the chip may happen when the antenna’s directivity is insufficient. (**b**) A new wireless interconnect architecture utilises digital coding metamaterials (purple regions) to modulate the carrier wave after the transmitter antenna. The modulated signals are directional, allowing one TX antenna to serve multiple receiver targets.

Figure 1(a) illustrates a simplified schematic diagram of an intra-chip wireless interconnect based on surface wave propagation. Similar to most wireless interconnects, the RF transmitter comprises a series of wireless transceiver modules, including a local oscillator that generates the high-frequency carrier wave signal. The input digital signal is sampled and converted by a digital-to-analogue converter (DAC) and then loaded on the carrier wave through a mixer or signal modulator^6–8^. The modulated carrier wave will undergo continuous changes in its amplitude, phase, or frequency in the time domain, depending on the input digital data and the modulation scheme. The time-varying signal is amplified and transmitted to the on-chip antenna, where it is broadcast to the receiver.

In conventional wireless interconnect architectures, digital information is modulated onto a carrier wave and transmitted via an on-chip antenna, making the directionality and transmission range predominantly determined by the antenna’s performance. However, most existing intra-chip wireless interconnects continue to suffer from low gain, limited directivity, and poor interference immunity, owing to stringent constraints on on-chip integration^9,10^. A schematic representation of a conventional intra-chip wireless interconnect is shown in Fig. 1(a), where a central transmitter antenna excites surface waves propagating along the dielectric silicon layers, radiating to eight surrounding receivers to form a broadcast-like communication mode. However, if the transmitted data is intended only for a single receiver, a significant portion of the EM energy radiated toward other regions of the chip remains underutilised, leading to inefficient power use and potential interference with neighbouring circuits. Typically, traditional wireless transmission architectures introduce additional medium access control methods to increase transmission bandwidth and control crosstalk, such as token-passing^11^, frequency division^12^, and code-division multiple access^13^. However, this usually increases transmission delay and hardware complexity and cost.

With the development of wireless communication technology and theory, metamaterials enable the control and modulation of electromagnetic waves beyond traditional antennas, introducing a new paradigm and enhancing engineering applications in wireless transmission systems. Among them, the digital-coding metamateiral leveraging discrete coding states with integrated diode, transistor and amplifier elements on unit cells to create reconfigurable and programmable electromagnetic responses^14,15^. Which can dynamically control electromagnetic wave propagation in both space and time domains^16–18^, with advanced features like beam-steering^19,20^, nonreciprocal radiation^21,22^ and self-adaptive control^23,24^.

Digitally encoded metamaterials can transform a digital coding sequence into a specific electromagnetic wave response, which changes the properties of the EM wave reflected or transmitted through the metamaterial. This process is very similar to the usage of a circuit modulator to encode digital information onto high-frequency carrier waves in traditional wireless transmission^25^. Therefore, the interaction of EM waves with the digital-coding metamaterials can be considered as a signal modulation process. Previous works^26,27^ have pioneered a novel wireless communication system using digital coding metamaterial for direction signal modulation. The modulation circuit system is vastly simplified, with only a programmable metamaterial and an FPGA used to control it. Early modulation metamaterial features a dual-state unit cell that supports simple modulation schemes, such as BFSK and ASK^27,28^, which enables a transmission data rate of 78.1 kbps at a carrier frequency of 3.6 GHz. Further progressions demonstrate more complex phase-shift keying (PSK), such as QPSK^29^ and 8-PSK^30^, using a varactor diode-loaded metamateiral to achieve a higher data rate of 6.14 Mbps. Work^31^ presents a modulation metasurface that can support multiple high-order modulation schemes, including 8-PSK and QAM. And a modulation data rate of up to 10 Mbps on a 4.25 GHz carrier wave. In summary, coded metamaterials have been shown to be sufficiently feasible and capable of supporting most commonly used wireless coding methods. Compared to traditional wireless transmission architectures, digital coding metamaterials can realise complex modulation schemes with only digital inputs, without the requirement for complex amplitude and phase-shifting circuits, which may help reduce the manufacturing complexity and cost of wireless communication systems.

On-chip metamaterials offer a promising solution for enhancing antenna performance and increasing intra-chip interconnection flexibility. In our previous work, an on-chip digital-coding metamaterial is proposed that can greatly enhance the beam-steering capability of on-chip dipole antennas^32^. This paper further advances this concept, introducing a multi-functional digital coding metamateiral capable of supporting multiple digital modulation schemes and dynamic beam-steering for intra-chip wireless transmission. The proposed metamaterial consists of three cascaded stages that generate phase shifts of $180^{\circ }, 90^{\circ },\, \hbox {and}, 45^{\circ }$ as electromagnetic waves propagate through it. This configuration enables precise phase manipulation and supports various modulation modes and coding patterns, including BPSK, QPSK, and 8-PSK. Furthermore, it introduces a hybrid operational mode, allowing simultaneous BPSK modulation and dynamic beam steering with a steering range $\pm 28^{\circ }$.

## Theory and design concept

### Concept of metamaterial modulated intra-chip interconnect

The EM surface wave can propagate inside the silicon substrate in either TE or TM mode. In our design, the proposed digital coding metamateiral is designed to modulate the propagation of a TE-mode surface wave on a silicon substrate^33,34^. This is mainly due to the fabrication of TE-mode antennas that can utilise metal contact/interconnect layers in most nanofabrication platforms, with superior compatibility. In comparison, TM wave propagation commonly requires excitation via TSV (Through Silicon Via) antennas or transducers, which necessitate complex fabrication procedures incompatible with standard CMOS processes and increase implementation difficulties^5,35^.

Figure. 1(b) presents the schematic design of an innovative intra-chip interconnect based on signal modulation through digital-coding metamaterial. The transmitter antenna directly receives and broadcasts an unmodulated carrier wave from the local oscillator, eliminating the need for traditional circuit components for modulation, such as DACs and mixers, leading to a great simplification of the transmitter circuit. Instead of performing modulation within the transmitter circuit, the modulation process occurs as the radiated surface wave propagates through the digital-coding metamaterial fabricated on the surface of the silicon substrate. The metamaterial is engineered to apply specific phase shifts to the carrier wave based on the input data. These phase shifts are designed to align with the mapping relations of standard phase-shift keying (PSK) schemes, such as BPSK, QPSK, and 8-PSK. By dynamically adjusting the phase shift values of the metamaterial in the time domain according to the input data, the proposed system can achieve a coding effect similar to that of conventional phase-shift modulation circuits.

**Fig. 2:**
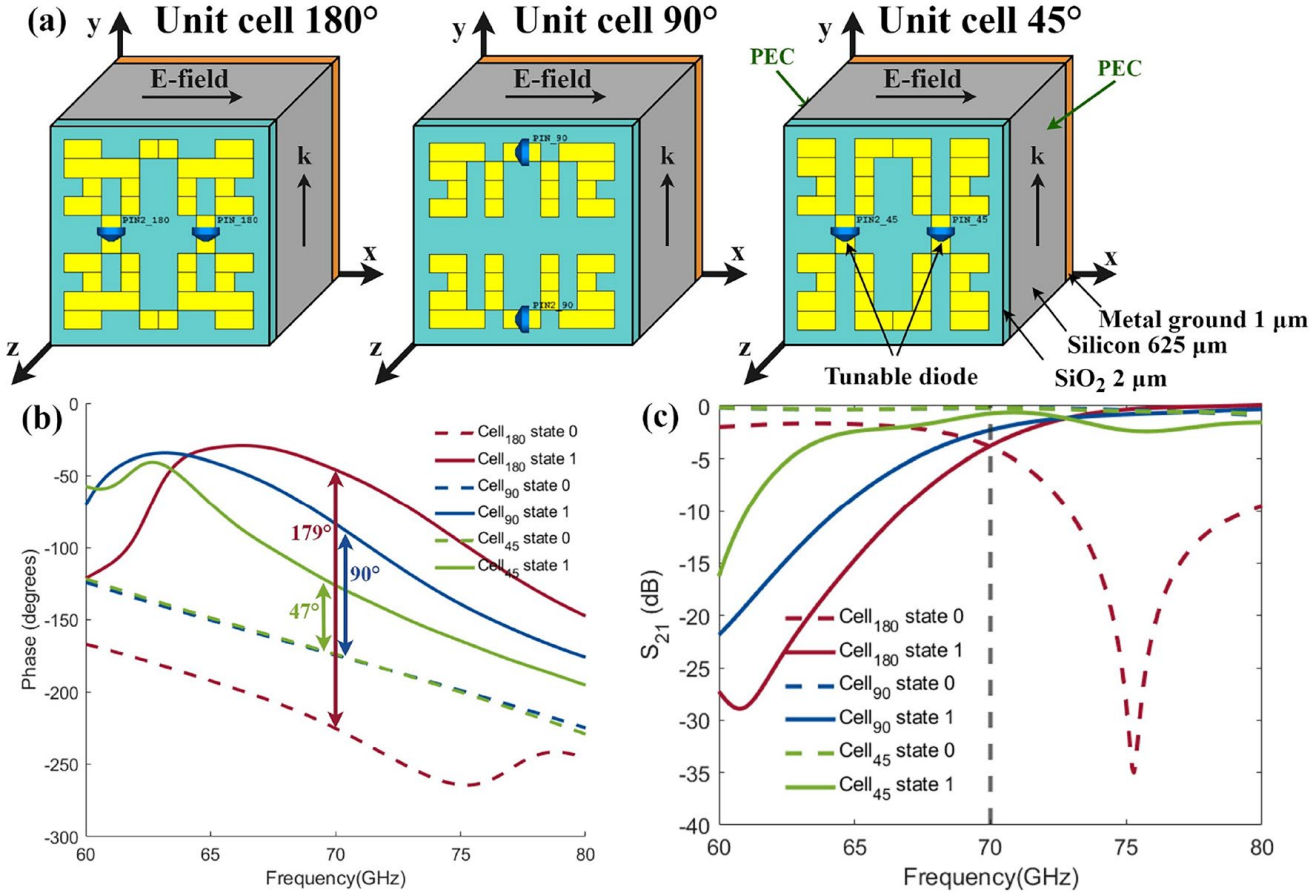
Design of three metamaterial unit cells for on-chip phase-shifting modulation. (**a**) Layout design of three unit cells: each cell is loaded with diode elements to achieve two switchable states with a $180^{\circ }, 90^{\circ }\,\hbox {and}\, 45^{\circ }$ phase difference. Simulated (**b**) phase and (**c**) amplitude response of the transmitted coefficient of the three unit cells under state’ 0’ and ’1’.

The metamateiral signal modulation provides the following advancement over the conventional approach. Firstly, modulation occurs only when the EM wave propagates through the metamaterial, ensuring that modulation targeted to a specific receiver does not affect other areas of the chip. This enables highly directional data delivery, reducing the risk of interference with other transmission channels. Secondly, the metamaterial enables more efficient use of the EM energy from the transmitter antenna by implementing spatial multiplexing within the limited transmission space of a silicon chip. As shown in Fig. 2(b), multiple sets of metamaterials can be configured to transmit different data sequences to distinct receivers, thereby vastly expanding the data throughput from a single transmitter. Furthermore, the proposed metamaterials are highly versatile, supporting multiple modulation schemes, including hybrid modulation and beam deflection functionalities. Each metamaterial unit can be independently controlled and coded, offering exceptional flexibility and reconfigurability for on-chip interconnects.

### Modulation principle

An unmodulated time-varying EM carrier with a frequency of $f_c$ generated by a TX antenna can be expressed as:

1 $$\begin{aligned} E_i(t) = A_i e^{j2\pi f_ct} \end{aligned}$$

As the EM wave propagates through the metamaterial. The EM wave interacts with the metallic structure of the metamaterial, resulting in a change in both the amplitude and phase components. This process can be summarised into a time-varying transmission coefficient *T*(*t*) multiplied by the carrier wave^29,31^.

2a $$\begin{aligned} E_t(t)= & T(t) \cdot e^{j2\pi f_ct} \end{aligned}$$

2b $$\begin{aligned} T(t)= & T_m(t) \cdot g(t),\ 0 \le t \le T_s \end{aligned}$$

2c $$\begin{aligned} T_m(t)= & A(t) e^{j \Phi (t)},\ 0 \le t \le T_s \end{aligned}$$

where g(t) serves as the pulse-shaping function during the symbol period $T_s$. $T_m(t)$ is the transmission coefficient of the message symbol with the amplitude and phase component of A and $\Phi$. To achieve a valid wireless modulation scheme, the amplitude and phase components added by the digital-coding metamaterial must be designed to match the coding requirements of constellation diagrams under the given input of digital information. For instance, under an ideal BPSK modulation, the $T_m(t)$ should be expressed as $1 \cdot e^{j 0 \pi }$ and $1 \cdot e^{j \pi }$ during the symbol period under a data input of ’0’ and ’1’. Where two states have the same amplitude component of 1, and two phases are separated by $180^{\circ }$. Similarly, the amplitude and phase components required by the QPSK and 8-PSK modulation are listed in Table 1.

**Table 1:** Transmission coefficient required for different phase shifting modulation schemes.

Modulation scheme	Transmission coefficient $T_m(t)$						
BPSK	$1 \cdot e^{j 0 \pi }$				$1\cdot e^{j \pi }$		
QPSK	$1 \cdot e^{j 0.25 \pi }$		$1 \cdot e^{j 0.75 \pi }$		$1 \cdot e^{j 1.25 \pi }$		$1 \cdot e^{j 1.75 \pi }$
8-PSK	$1 \cdot e^{j 0.25 \pi }$	1 $\cdot e^{j 0.5 \pi }$	$1 \cdot e^{j 0.75 \pi }$	$1 \cdot e^{j \pi }$	$1 \cdot e^{j 1.25 \pi }$	$1 \cdot e^{j 1.5 \pi }$	$1 \cdot e^{j 1.75 \pi }$

## Metamaterial design

**Fig. 3:**
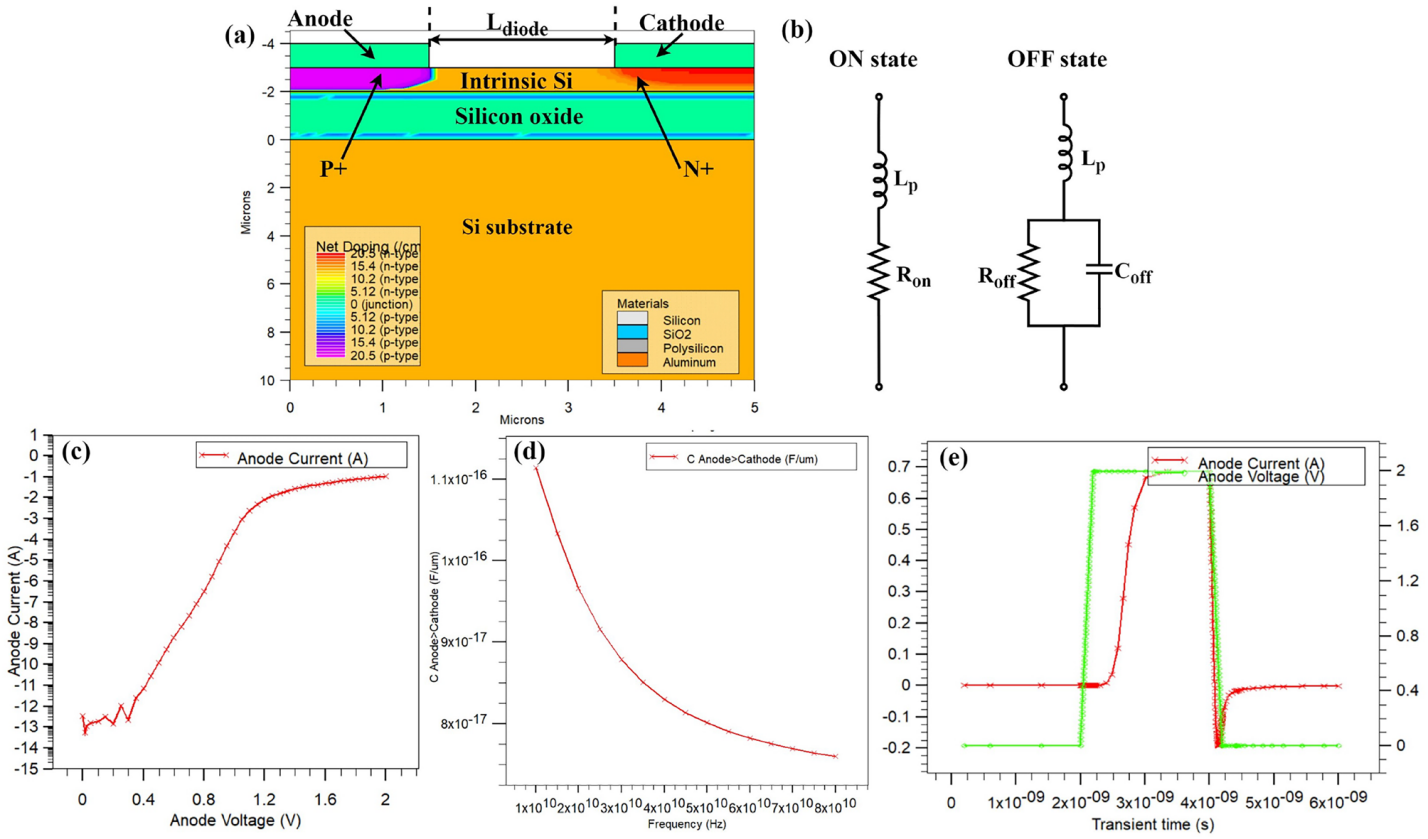
(**a**) The cross-section net doping diagram of the PIN junction that is used as the switching component for the unit cells. (**b**) Simplified circuit model for PIN junction under ON and OFF states^36^. (**c**) I-V characteristic of the PIN diode with a forward bias voltage from 0 to 2 V simulated with the Silvaco TCAD. (**d**) Simulated junction capacitance vs. frequency through small AC signal analysis. (**e**) Transient response of the PIN diode against a 2 ns square voltage pulse on the anode.

### Design of phase-shifting coding unit-cell

To validate the phase shift values required in Table 1, we proposed three metamaterial unit-cell designs that convert digital input values of 0 and 1 into specific phase shifts of $45^{\circ }, 90^{\circ },\hbox {and}\, 180^{\circ }$, laying the groundwork for the realisation of different PSK schemes. The design of the three unit cells is presented in Fig. 2(a). The metamaterial unit adopts a pixelated design; each unit cell is composed of a $5\times 5$ array of square pixels encoded as a 25-bit binary string. Each pixel can be defined as either ’1’ or ’0’, representing the presence or absence of metal coverage within its area. The bit map is mirrored twice to form a complete unit cell containing 100 small pads. The optimisation is performed through the CST-MATLAB API using the Genetic Algorithm to maximise the achievable phase shift around 70 GHz, with similar processes presented in works^32,37,38^. The pixelated architecture enables flexible reconfiguration from base structures to realise different phase-shift responses by introducing tunable elements at specific pixels.

Each phase-shifting unit cell includes a ’critical pixel’ that incorporates a PIN diode loaded onto a selected pixel to establish connectivity between the upper and lower (or left and right) parts of the unit cell. This design allows each unit cell to exhibit two distinct resonance states at different frequencies. The two operational states of the unit cell can be expressed as a binary bit in either ’0’ or ’1’, based on the switching state of the PIN diode. The state’ 0’ present diode is switched OFF, disconnecting the two parts of the unit cell. In this state, the unit cell resonates in a segmented manner at a higher frequency. State’ 1’ present diode is switched ON, creating a conductive interconnection between the two parts. In this state, the unit cell resonates as a unified structure at a lower frequency. The operating band of the phase-shifting unit is set between the resonance frequencies of ’0’ and ’1’ states, where a large phase difference but only a small amplitude difference in the transmission coefficient value between the two states. All three unit cells in Fig. 2(a) have a sidelength of 660 $\upmu \hbox {m}$, while different phase-shift values are achieved by varying the pixel pattern and the position of the PIN diode.

PIN diodes offer high-switching speed and effective isolation under the ’OFF’ state due to the intrinsic layer between the P and N layers, which are widely used as switching components in previous digital-coding metamaterials to achieve dynamic EM response and signal modulation^26,40,41^. For an on-chip digital-coding metamaterial, the SOI PIN diode can serve as a tunable component, easily integrated with the metallic unit-cell pattern and fully compatible with standard CMOS fabrication processes. Fig. 3(a) presents the cross-section map of the SOI PIN diode simulated with Silvaco TCAD; the P+ and N+ regions are doped with ion implantation with a concentration of $1 \times 10^{19}\,\hbox {atoms/cm}^{3}$ and followed by a short anneal. The intrinsic region is lightly doped with phosphorus with a concentration of $1\times 10^{14}\,\hbox {atoms/cm}^{3}$. The anode and cathode are metal patches extended from the metallic structures of the unit cell. The length of the intrinsic region $L_{\text {diode}}$ is $2 \,\upmu \hbox {m}$, and the thickness of the $\hbox {SiO}_{2}$ and poly-silicon layers is $2\,\upmu \hbox {m}$ and $1\,\upmu \hbox {m}$.

Full-wave simulation of the phase-shifting unit cells performed in CST. The unit cells are placed and simulated on a multi-layer silicon substrate comprising several metal and insulator layers, including a $1\,\upmu \hbox {m}$ metallic ground layer, a $625\,\upmu \hbox {m}$ High-Res (50 $\Omega$.cm) thick silicon substrate and a $2\,\upmu \hbox {m}$ thick $\hbox {SiO}_{2}$ insulating layer between the pattern and substrate. The substrate condition is sufficient to excite a $TE_{1}$ surface wave propagation at the designed operation frequency (70 GHz) of the unit cells^39^. Perfect electric boundary conditions (PEC) are applied in the $\pm x$ plane to simulate the field distribution of TE mode surface wave propagation inside the substrate generated by the on-chip dipole antenna. The remaining planes are set to the PML boundary condition. Two plane wave ports are defined on the $\pm y$ plane with an EM wave propagating along the positive *y* direction. The RCL parameters of the PIN diode in the ’ON’ and ’OFF’ states are loaded into the element design to simulate unit-cell characteristics in the ’0’ and ’1’ states.

During EM simulation, the PIN junction was modelled using a simplified equivalent circuit under ’ON’ and ’OFF’ states, as shown in Fig. 3(b)^36^. Under forward bias (ON state), the PIN junction can be modelled as a small resistance ($R_{on}$) in series with a parasitic inductance ($L_{p}$). Under 0 V or reverse bias (OFF state), the junction is modelled as a large resistance ($R_{off}$) connected in parallel with the junction capacitance ($C_{off}$) due to the presence of the intrinsic layer between the anode and cathode, and the combination is connected in series with the parasitic inductance ($L_p$). The DC I-V response of the PIN diode is presented in Fig. 3(c) with a positive voltage bias applied on the anode from 0 to 2 V. The PIN junction provides a very large series resistance $R_{off} > 5000 \Omega$. When the forward-biased voltage reaches 2 V, the diode is fully switched on, and the free carriers are injected into the intrinsic region, which remarkably reduces the resistance $R_{on}$ to around 6 $\Omega$. The junction capacitance $C_{off}$ under the ’OFF’ state was extracted from small-signal AC analysis performed at a 0.01 V bias. Figure 3(d) plots the junction capacitance between anode and cathode from 10 to 80 GHz. The junction has a capacitance value of $7.7\times 10^{-17}$ F/$\upmu \hbox {m}$ at 70 GHz. A PIN junction width of $60\,\upmu \hbox {m}$ will give a total capacitance of 4.62 fF.

The parasitic inductance ($L_p$) primarily originates from bonding wires and the package of the diodes. As the PIN junction is closely integrated with the metamaterial structure, it eliminates the need for external wire bonding. Therefore, the parasitic inductance $L_p$ can be less than 1 pH if only considering the contact vias between the junction and the metamaterial patch. Given the potential presence of parasitic components during fabrication, a quantitative analysis of the maximum tolerable parasitic components at the PIN junction has been conducted using EM simulations. The tolerance threshold was defined as a deviation of $22.5^{\circ }$ in phase shift and 3 dB in amplitude shift from the ideal modulation point, which can result in complete failure of a single 8-PSK modulation point. That upper limit for the junction capacitance $C_{off}$ is 32 fF,$L_p$ is 20 pH and $R_{on}$ is 12 $\Omega$.

The modulation rate of the unit cell depended on its switching speed. Figure 3(c) presents the transient simulation of the PIN diode against a 2-nanosecond square pulse applied to its anode. The diode exhibits a fast turn-on time and a short recovery time in the nanosecond range, which would theoretically support a modulation rate of more than 500 MHz. Additionally, the SOI PIN diode structure is also widely used as a carrier-injection optical modulator in silicon photonics circuits. The reported modulation speed of the diode can be further expanded to 10 - 20 Gbps with a pre-emphasised driving signal and a proper choice of DC offset voltage^42–44^, which is sufficient to meet the high bandwidth requirements for transmission at millimetre-wave band.

Figure 2(b) presents the simulated phase response of the transmission coefficient. The dotted lines indicate the phase response of ’0’ states unit cells where the diode is switched OFF. The solid lines indicate the ’1’ states where the diode is switched on. At an operation frequency of 70 GHz, the three types of phase-shifting unit cells can generate phase differences of $47^{\circ }, 90^{\circ },\hbox { and } 179^{\circ }$. Unlike the phase response, the amplitude of the transmission coefficient should remain unchanged when switching between ’0’ and ’1’ states to ensure pure phase modulation. However, for resonance-type metamateiral unit cells, the amplitude and phase response of the unit cells are strongly coupled near the resonance frequency. This means the change in the phase component will inevitably affect the amplitude. Therefore, we have adopted a design approach that ensures the amplitude component of each unit cell is aligned on a similar level in both switching states. Figure 2(c) plots the amplitude response of the three unit cells against frequency. Although the corresponding curves for each cell in the ’0’ and ’1’ states are vastly different, all curves reach a similar amplitude level near the frequency of 70 GHz.

### Design of cascaded digital-coding metamateiral

**Fig. 4:**
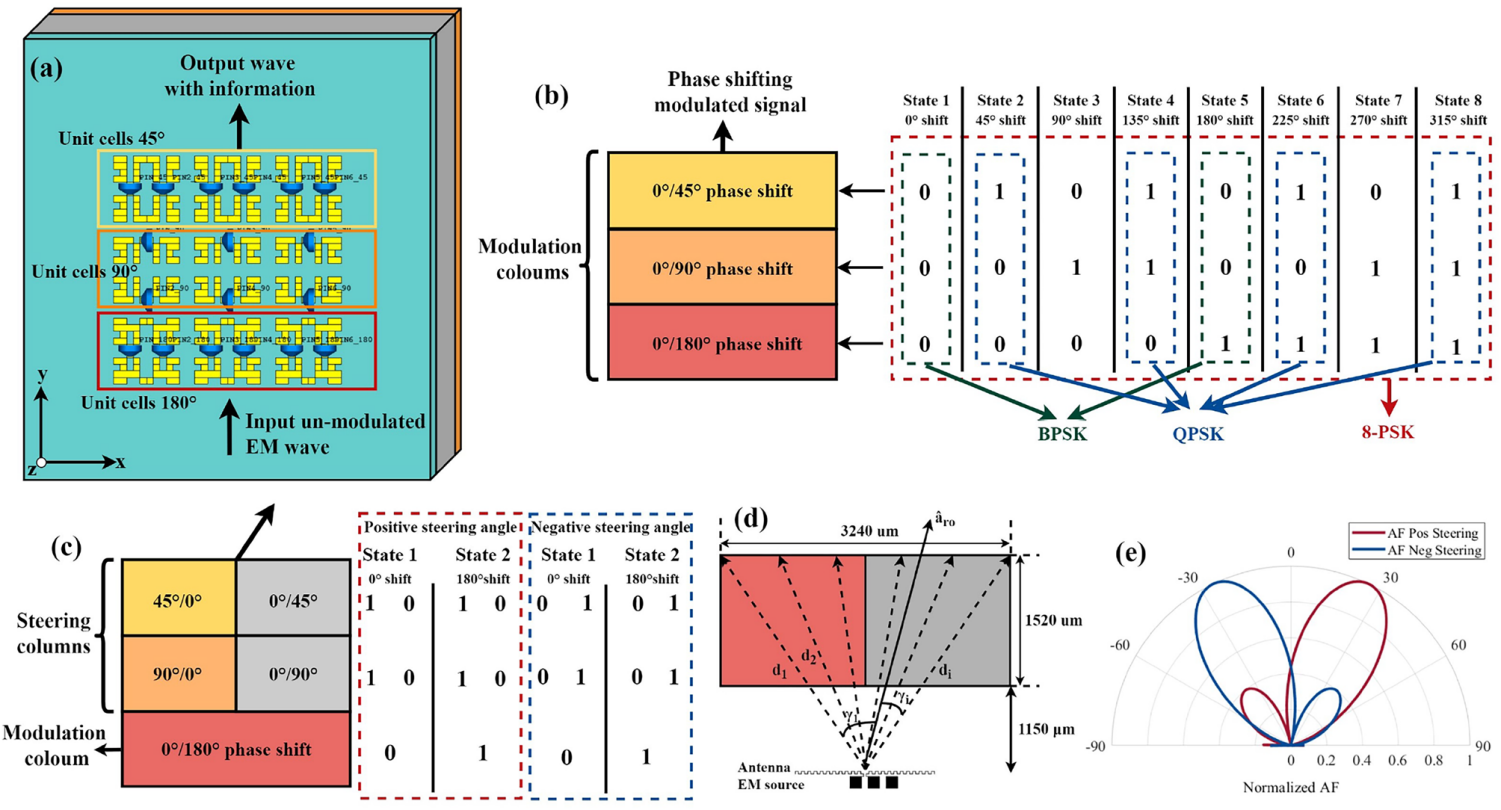
(**a**) Schematic design of the modulation metamateiral formed by three columns of unit cells that can provide a $180^{\circ }, 90^{\circ } \hbox { and } 45^{\circ }$ phase shift. (**b**) The modulation mode of the digital coding metamateiral is when all three columns are used for phase-shift keying, and the phase-shift table can be generated from different input bits to achieve BPSK, QPSK, and 8-PSK modulation schemes. (**c**) Beam-steering and modulation mode, where the bottom $180^{\circ }$ column was used for BPSK modulation, and the top two columns were unevenly switched for beam-steering. (**d**) Ray model analysis from the EM source for array factor calculation. (**e**) The calculated AF based on ray-model analysis presents a beam deflection angle of approximately $\pm 27^{\circ }$.

Based on the design of the proposed phase-shifting coding unit cells, we propose a digital-coding metamaterial for on-chip phase modulation. As shown in Fig. 4(a), the metamateiral has a three-stage cascaded structure composed of three columns of phase-shifting unit cells that can provide a phase shift of $180^{\circ }, 90^{\circ }\hbox { and } 45^{\circ }$, respectively, with an inter-column spacing of $200\,\upmu \hbox {m}$ to reduce the inter-column coupling, which can affect the modulation accuracy. As the intra-chip EM wave propagates through the metamateiral, it will interact with the three columns of cells in sequence, which would theoretically cover a phase-shifting range of $315^{\circ }$. The switching states of the unit cells in each column will affect the phase of the output electromagnetic wave. By applying a time-varying digital signal across the diodes of each of the unit cells, it would be possible to modulate the input EM carrier wave in real-time.

The proposed metamateiral features two operation modes for different intra-chip transmission requirements. The first mode shown in Fig. 4(b) is the modulation mode, in which all three unit-cell columns are utilised for data modulation. The modulation mode accepts a three-bit digital data input, with each bit controlling the switching state of one column of unit cells. Fig. 4(b) shows the mapping between the 3-bit data input and the output phase shift. The metamateiral can cover a phase shift range from $0^{\circ }\,\hbox {to}\, 315^{\circ }$ as the 3-bit input increments from ’000’ to ’111’. It can also support up to 8 switchable modulation states with a minimum modulation step size of $45^{\circ }$. Based on the digital-phase mapping, the proposed modulation metamateiral can theoretically support multiple modulation schemes, including BPSK, QPSK and 8-PSK in binary coding sequences.

The second operation mode offers a hybrid modulation and steering capability, as shown in Fig. 4(c). In this mode, only the bottom column with a $0^{\circ }/180^{\circ }$ phase shift is used for modulation, while the top two phase-shifting columns steer the beam of the on-chip antenna. In this mode, the metamaterial can perform BPSK modulation on the carrier wave and deflect the modulated beam over a range to meet transmission requirements in different directions. Metamaterials can deflect the transmission direction of the EM wave by constructing a gradient phase distribution within their structure^19,45^. By unevenly switching the left and right halves of the $45^{\circ }\,\hbox {and}\, 90^{\circ }$ columns, the metamaterial creates a non-symmetric phase shift when the EM wave propagates through its left and right halves. The wavefront of the EM wave will be deflected toward the direction with a lower phase shift. The modulation and steering mode requires a total of 5 control bits: 1 bit for BPSK modulation and 4 bits to control the beam steering direction and angle.

The radiation pattern under the steering mode can be estimated using the EM ray model and the Array Factor calculation at farfield. The EM wave radiated by the EM source is approximated as multiple EM rays. Each ray will travel into the metamateiral region along a different path length and with a different phase shift. The calculation of the array factor (AF) characterises the far-field pattern as the sum of contributions from each ray with the formula below^46,47^.

3 $$\begin{aligned} AF = 1+\sum _{i} e^{jk_rd_i\text {cos}(\gamma _i)}, \end{aligned}$$

Where $d_i$ represents the path length of each EM ray, and $\gamma _i$ represents the interior angle between each ray and the observation direction $\hat{a}_{ro}$. The simplified metamateiral ray model is presented in Fig. 4(d), with EM rays radiated from the MDA (EM source) travelling into the beam-steering metamateiral regions ($90^{\circ }\,\hbox {and}\, 45^{\circ }$ steering columns), which have dimensions of $3420 \times 1520\,\upmu \hbox {m}$. The beamsteering columns can provide a maximum phase difference of $135^{\circ }$ (2.35 rad) as the EM wave passes through the left and right half planes of the metamaterial. This is added to the phase component of the ray expression. The calculated radiation pattern on the top azimuth plane ($-90^{\circ }\,\hbox {to}\, +90^{\circ }$) is plotted in Fig. 4(e) by multiplying the AF by the element factor of a dipole antenna^46^. The calculated result indicates a maximum beam deflection angle of around $27^{\circ }$. This can contribute to a total beam deflection range of $\pm 27^{\circ }$ with a configuration under positive and negative steering angles.

In conclusion, the digital coding metamaterial we proposed features functional multiplexing by encoding the same cell in different forms. The modulation mode can provide the highest data rate, supporting modulation schemes up to 8-PSK. The hybrid modulation and beam steering mode can simultaneously support a BPSK modulation scheme and a beam steering range of $\pm 27^{\circ }$. The same device can be a flexible switch to ensure a high data rate or high directivity.

## Evaluation and results

**Fig. 5:**
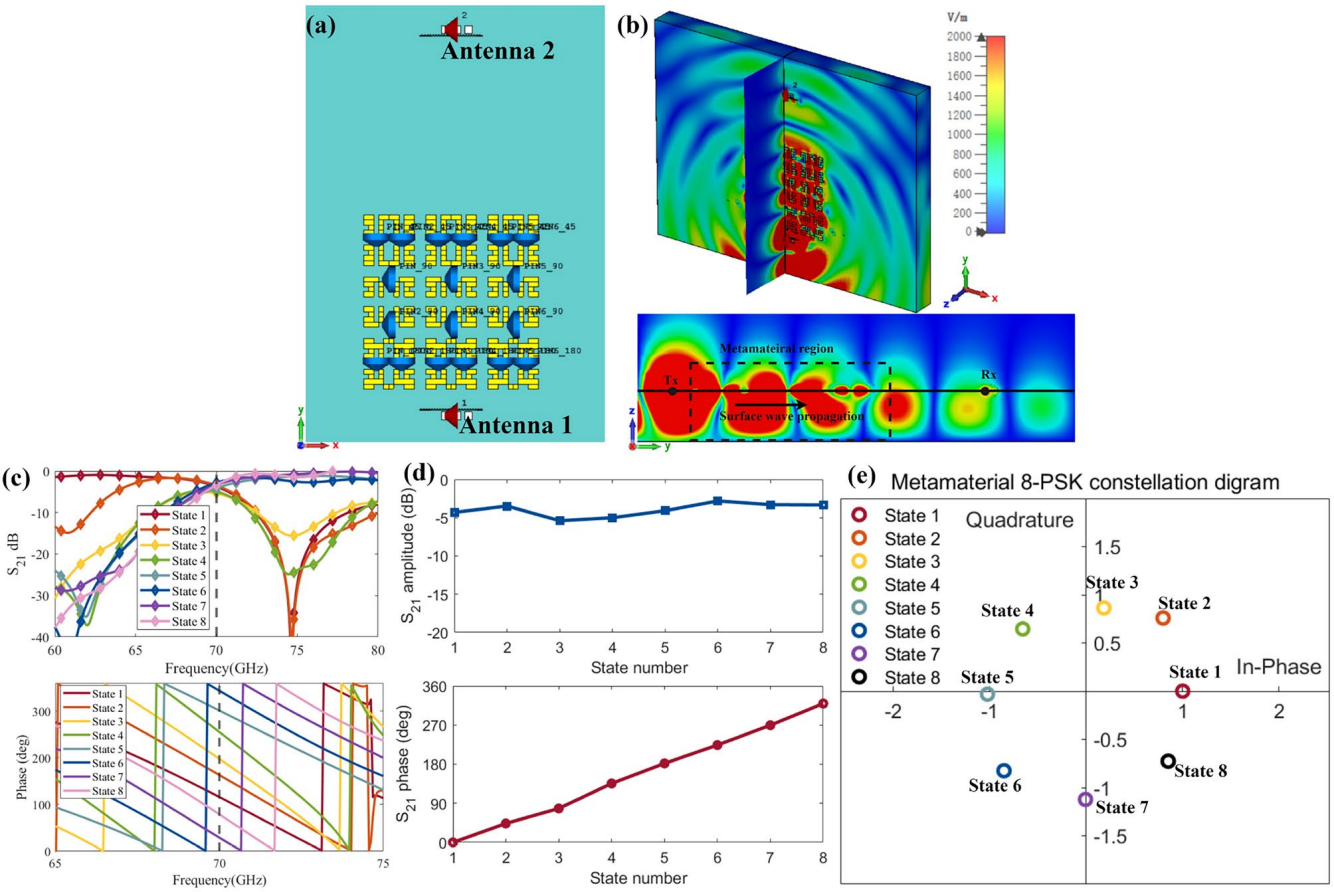
Simulation of the proposed digital-coding metamaterial under modulation mode. (**a**) Simulation setup of modulation mode with three columns of phase-shifting unit cells placed between an MDA pair and configured with digital input from ’000’ to ’111’. (**b**) The electric field distribution as the EM wave propagates through the metamaterial on the horizontal and vertical planes. (**c**) Amplitude and phase curves of the transmission coefficient ($S_{21}$) vs. frequency from state 1 to 8. (**d**) Normalised amplitude and phase component of transmission coefficient at 70 GHz with data input from states 1 to 8. (**e**) The mapping constellation diagram of the 8-PSK modulation based on the simulated transmission coefficient.

**Fig. 6:**
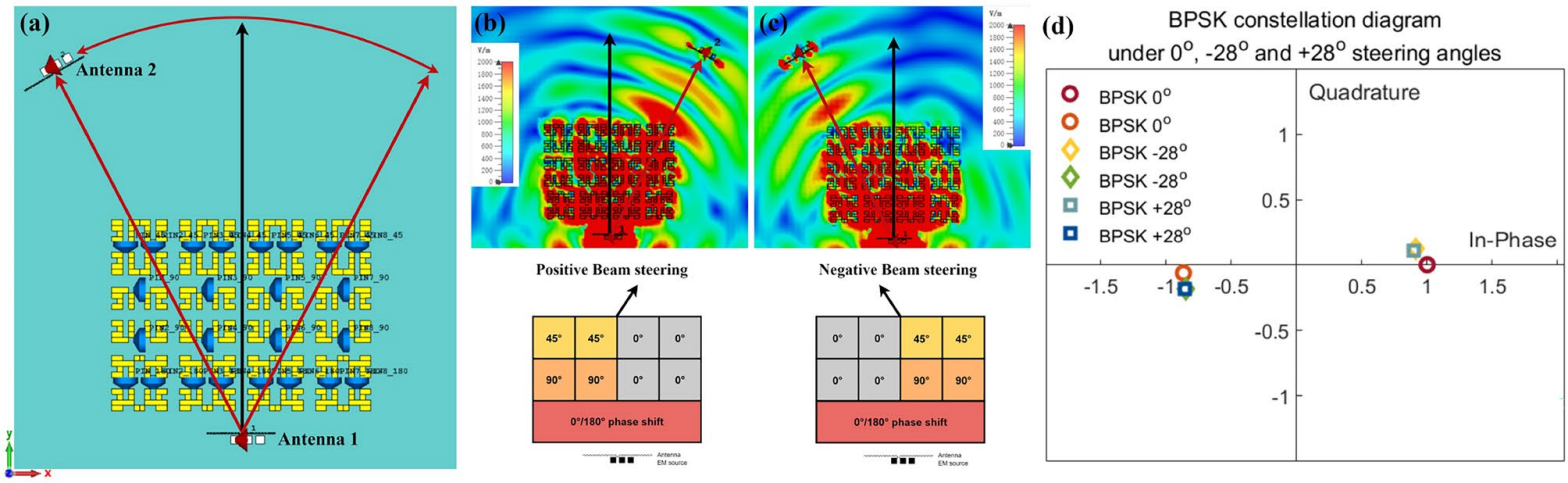
Simulation of the proposed digital-coding metamaterial under hybrid modulation and beam steering mode. (**a**) Simulation setup for beam steering test, where the receiver antenna 2 is rotated around the centre of antenna 1 to receive a signal under different steering angles. (**b**) Simulated E-field distribution under a $+28^{\circ }$ beam deflection angle and (**c**) $-28^{\circ }$ deflection angle. When the top two columns of unit cells are unevenly switched to create a phase gradient. (**d**) Mapping constellation diagram of BPSK modulation under $0^{\circ }, -28^{\circ }\,\hbox {and}\, +28^{\circ }$ beam deflection angle.

Figure 5(a) presents the simulation setup of the metamaterial under modulation mode. The metamateiral is placed between a pair of on-chip meandering dipole antennas (MDAs) with a separation distance of 5 mm. The MDA has an $S_{11}$ value of -22 dB at 70 GHz and two main lobes positioned upwards and downwards direction with a maximum gain of 3.78 dBi^32^. Antenna 1 acts as an EM source, generating an EM wave incident perpendicularly on the digital-coding metamaterial. Figure 5(b) plots the EM field distribution as the EM wave propagates through the metamaterial on the horizontal (XY) and vertical (YZ) planes. It can be clearly observed that the majority of the energy is radiated as a surface wave in the horizontal plane. As the EM wave propagates through the metamaterial, a portion of its energy is attenuated due to EM resonance, accompanied by a phase delay, before it is captured by antenna 2. Three unit-cell columns of the unit cell are coded with a three-bit input ranging from ’000’ to ’111’, corresponding to States 1 to 8 in Fig. 4(b).

Figure 5(c) presents the simulated transmission coefficients ($S_{21}$) for each of the eight modulation states. The plot reveals noticeable phase variations in the transmitted signals across the states, while the amplitude remains relatively consistent, varying within a small range of -25 to -27 dB at 70 GHz. This suggests a relatively small amplitude fluctuation during the phase modulation process. However, all states exhibit increased signal attenuation at 70 GHz, indicating a clear insertion loss resulted from the EM resonance of the metamaterial.

Figure 5(d) presents the normalised amplitude and phase component of the transmission coefficient from states 1 to 8 at 70 GHz. The metamaterial exhibits relatively small amplitude fluctuations and a linear progression of phase-shift values from state 1 to state 8. The coding metamaterial exhibits a maximum frequency shift of from $0^{\circ }\,\hbox {to}\, 319.5^{\circ }$ and an amplitude fluctuation of 2.57 dB. Additionally, there is an average insertion loss of around 4 dB when the metamateiral is placed on the signal propagation path. Compared to the ideal phase-shifting modulation, the metamaterial is insufficient to achieve a perfectly flat amplitude response and a uniform phase shift of $45^{\circ }$ at each step; however, the simulation results still confirm the feasibility of achieving 8-PSK modulation.

Figure 5(e) presents the simulated constellation diagram where the amplitude and phase components of eight operating states are mapped on the IQ plane. Where the amplitude and phase component of the state 0 with a ’000’ data input is set as the reference point, which is defined to have a unit amplitude value of 1 and a $0^{\circ }$ phase shift. The amplitude and phase components of the remaining seven modulation points are mapped to the diagram. The result constellation diagram shows a relatively good match with the ideal 8-PSK modulation. The error vector magnitude (EVM)^48^ evaluates the accuracy of the constellation diagram by comparing the difference in position between the ideal constellation point and the actual point. The 8-PSK constellation diagram of the metamaterial has an average EVM of 13.3% and a maximum EVM of 22.8% at the 010 point. This result will not affect the implementation of 8-PSK modulation, but it will compress the noise margin between different constellation points, resulting in weaker noise immunity.

Figure 6(a) presents the simulated setup of the proposed metamaterial under hybrid-beamsteering and modulation mode. Each column contains four unit cells to ensure a higher beam-steering angle. The black arrow indicates the main lobe direction of the transmitter antenna. The receiver antenna 2 is rotated around the centre of the transmitter antenna 1 to receive the signal under different beam steering angles with a constant separation distance of 5 mm. The top two columns of the metamaterial are unevenly switched to create a $135^{\circ }$ phase difference that steers the antenna beam, and the bottom column is used to generate a $180^{\circ }$ phase shift for BPSK modulation.

Figure 6(b) and (c) illustrate the electric field (E-field) distribution for two metamaterial configurations, corresponding to positive and negative beam-steering angles. The measured steering angle is approximately $28^{\circ }$, slightly exceeding the calculated value. To validate signal reception under beam steering, antenna 2 was rotated to the corresponding angle to capture the BPSK-modulated signal. Fig. 6(d) presents the constellation diagram of the BPSK modulation for steering angles of $0^{\circ }, -28^{\circ },\,\hbox {and}\, +28^{\circ }$. When compared to the ’standard’ BPSK constellation without beam steering, only a minor phase shift of approximately $10^{\circ }$ is observed at the $\pm 28^{\circ }$ steering angles. This phase shift is likely caused by the partial activation of the $45^{\circ }\,\hbox {and}\, 90^{\circ }$ phase-shifting unit cells. However, the observed phase deviation remains within acceptable limits for BPSK modulation, confirming the feasibility of simultaneous modulation and beam steering.

## Wireless channel characteristics

### Simulation setup

**Fig. 7:**
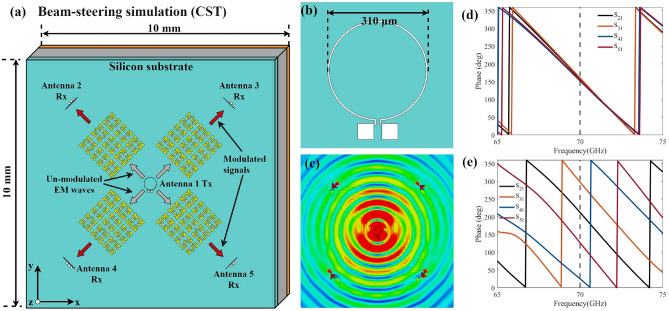
Simulation of multi-target modulation analysis. (**a**) Simulation setup of the multi-target modulation, the centre Tx antenna is placed in the middle of the chip, and four receiving dipoles are distributed on the four corners of the chip. Four digital coding metamaterials are used to modulate the receiver antenna in each direction. (**b**) Design of the transmitter ring antenna and (**c**) its radiation pattern on the E-field plane. (**d**) The phase shift of $S_{21}$ to $S_{51}$ when four metamaterials are (d) un-modulated and (**e**) modulated with $90^{\circ }$ out of phase.

Time-domain simulations were conducted to evaluate the modulation capabilities of the proposed digital-coding metamaterial under realistic intra-chip interconnect conditions. The simulation setup for multi-target modulation is illustrated in Fig. 7(a). A 70 GHz patch-ring antenna is positioned at the centre of the chip, acting as the wireless transmitter. Figure 7(b) presents the design of the on-chip ring antenna, which consists of a metallic ring with a diameter of $620\,\upmu \hbox {m}$ and two metal patches for signal feeding. Figure 7(c) presents the E-field radiation pattern of the ring antenna, demonstrating near-omnidirectional surface-wave radiation in the horizontal plane and consistent phase shifts across all receiving antennas in the absence of metamaterial modulation, thereby providing an unbiased baseline for evaluating modulation effects. The transmitter antenna is surrounded by four digital-coding metamaterials that respond to provide modulation to each receiver antenna. The transmitter antenna in the middle radiates an unmodulated carrier wave towards all directions. As the EM wave passes through the digital-coding metamaterials, it acquires a phase shift proportional to its coding state and is then captured by the receiver antenna.

**Fig. 8:**
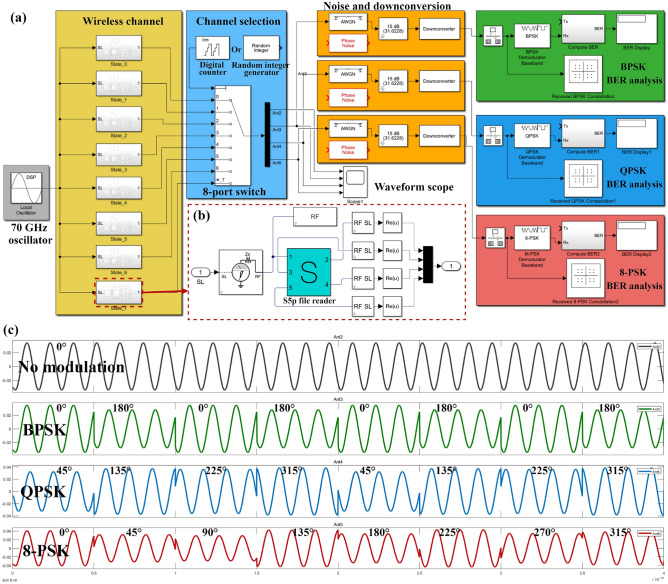
Simulation setup of time-domain transmission analysis in Simulink. (**a**) The Simulink block diagram for wireless modulation simulation contains five main sections: carrier wave generation, wireless channel simulation, channel selection, noise and downconversion, demodulation, and BER analysis. (**b**) Zoomed in on the block diagram of the wireless channel, the S-parameter block (highlighted in blue) reads one touchstone file from the multi-target modulation simulation from CST. Port 1 accepts the input carrier wave, and ports 2-5 output the received signal from antennas 2-5, which contains the amplitude and phase information after modulation by the digital-coding metamaterial. (**c**) The output of the waveform scope (Without noise) at the receiver antenna 2, 3, 4, and 5 indicates an un-modulated, BPSK, QPSK, and 8-PSK modulated waveform from top to bottom.

Four receiver MDAs are placed at the corners of the chip, labelled from 2 to 5. Each antenna is oriented such that its main lobe points toward the central transmitter antenna. The transmitter-receiver (Tx-Rx) separation for all receiver antennas is maintained at 5.65 mm, ensuring uniform performance evaluation across all channels. Figure 7(d) presents the received phase shift of the four antennas, where all four metamaterials are un-modulated. The same level of phase shift can be observed on each receiver antenna due to the same Tx-Rx separation distance. Since the four receiving antennas and the metamaterial are spatially dislocated by 90 degrees, each antenna can be independently modulated without affecting the others. For comparison, Figure 7(e) presents the case where ’001’, ’011’, ’101’, and ’111’ coding is applied to metamaterials 1 to 4. The signals received by the four antennae are $90^{\circ }$ out of phase with each other.

The Simulink is used to evaluate a time-domain modulation and demodulation process with the metamateiral, illustrated in the block diagram in Fig. 8(a). The simulation process is divided into five sections, each marked with a different colour. The process begins with a local oscillator generating a 70 GHz carrier wave, which is then distributed across eight wireless channel blocks. Each block corresponds to one of the eight operational states of the digital-coding metamaterial in modulation mode. Figure 8(b) provides a detailed view of the internal structure of a single wireless channel block. Each block contains an S-parameter module, highlighted in blue, which reads S-parameter Touchstone files generated by the CST multi-target modulation analysis (as shown in Fig. 7(a)). The carrier wave is fed into port 1, representing the transmitter antenna, while ports 2 through 5 output modulated waves corresponding to the four receiver antennas. Each wireless channel outputs varying phase and amplitude components based on the coding applied to the metamaterials. The outputs from the eight wireless channel blocks are connected to an 8-port switch controlled by a digital counter or a random integer generator, which dynamically selects the final output based on time-varying input data, simulating the modulation behaviour of the metamaterials. The modulated signals received by antennas 3, 4, and 5 are then processed with an additive white Gaussian noise (AWGN) or phase noise channel, amplified and downconverted. Finally, the signals are demodulated using a standard phase-shifting demodulator, and bit-error-rate (BER) analysis is performed to evaluate system performance.

**Fig. 9:**
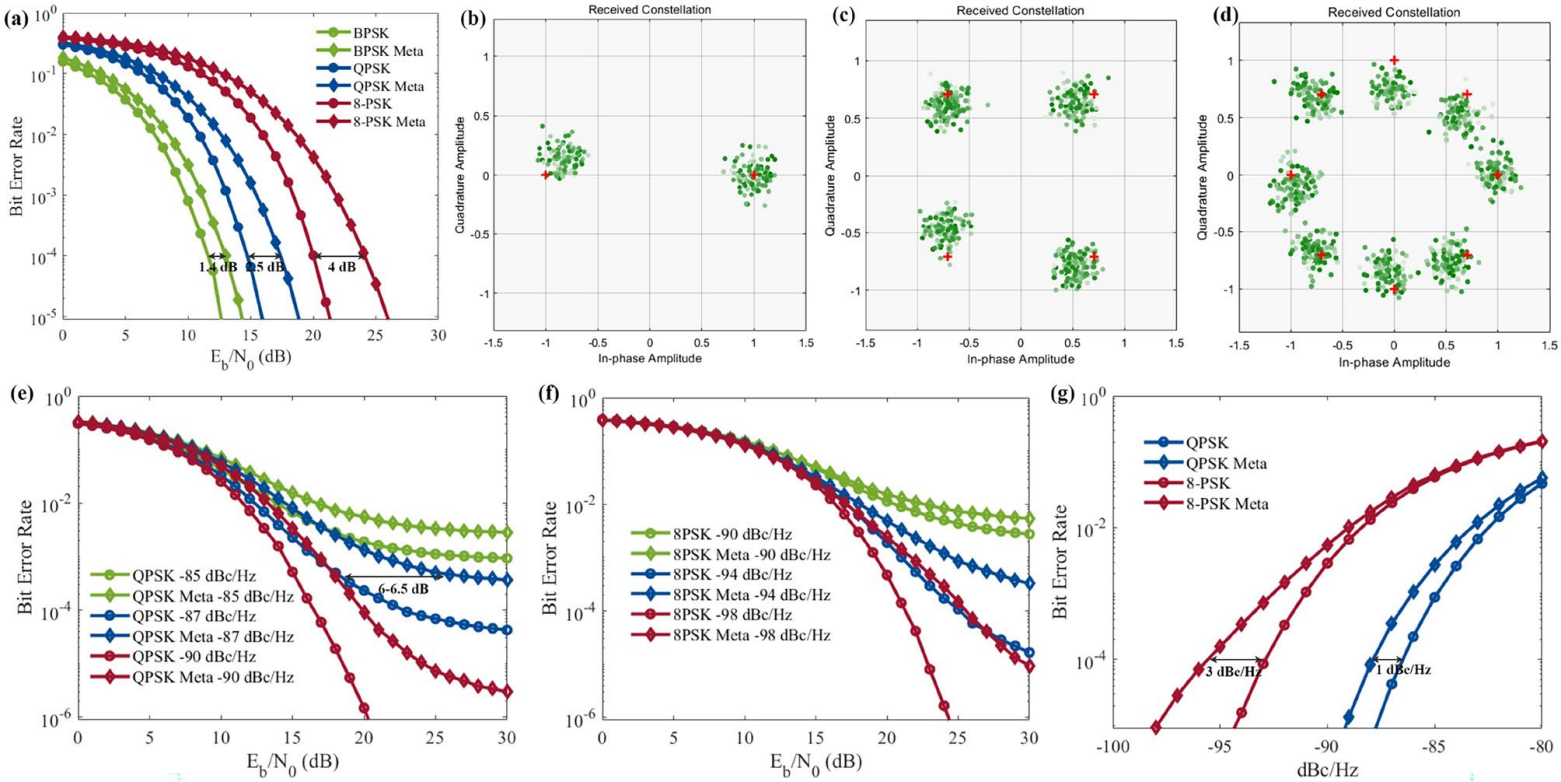
(**a**) Bit error rates (BERs) of BPSK, QPSK, and 8-PSK modulation achieved by metamateiral modulation compared with the ideal modulation schemes under different AWGN noise levels. The received constellation diagram for (**b**) BPSK, (**c**) QPSK and (**d**) 8-PSK modulation schemes under a signal-to-noise ratio ($E_b/N_0$) of 20 dB. BER of (**e**) QPSK and (**f**) 8-PSK modulation achieved by metamaterial compared with ideal modulation under different hybrid AWGN and phase noise levels. (**g**) BER of QPSK and 8-PSK modulation schemes under different phase noise levels.

Fig. 8(c) illustrates the output of the waveform of the 2, 3, 4 and 5 at the waveform scope as the digital counter connected to the 8-port switch increments from 0 to 7. Among them, antenna 2 outputs a stable sine waveform with no significant amplitude or phase variations, serving as a baseline for comparison. In contrast, the waveforms from antennas 3, 4, and 5 exhibit clear time-dependent phase variations corresponding to their respective modulation schemes. Antenna 3 demonstrates a distinct BPSK-modulated waveform, with an in-phase signal during timeslots 1, 3, 5, and 7 relative to antenna 2, and a $180^{\circ }$ out-of-phase signal during the remaining timeslots. Antennas 4 and 5 produce waveforms corresponding to QPSK and 8-PSK modulation with a phase shift of roughly $90^{\circ }\,\hbox {and}\, 45^{\circ }$ per step, respectively. It is worth noting that slight amplitude variations are observed during phase-shifting modulation. These variations are attributed to amplitude fluctuations during switching between operational states in Fig. 5(d). The impact of these fluctuations is further analysed in the BER evaluation.

### BER analysis

Bit-error-rate (BER) analyses were performed to evaluate the noise-resistance performance of the wireless channels using metamaterial modulation, compared with ideal phase-shift keying modulations. A sequence of randomly generated integers ($5 \times 10^6$) was used as the input of the switch. Additive white Gaussian noise (AWGN) is added to the wireless channel to simulate the intra-chip transmission noise. In the BER analysis blocks in Fig. 8(a), the received signals are decoded by a perfect PSK decoder, and the demodulated data are compared with the original data sequence to calculate the BER. Figure 9(a) plots the BER rate of the metamaterial modulation under BPSK, QPSK and 8-PSK compared with the ideal PSK modulation. The analysis revealed that metamaterial-based modulation consistently exhibits higher BER compared to ideal modulation schemes, with the performance gap varying across different signal-to-noise ratio (SNR) regions. The difference can be analysed in three stages. When $E_b/N_0 > 5$ dB, the wireless channels for both the metamaterial and ideal modulations experience significant noise interference. In this range, the BER is high for both systems, and no substantial performance difference is observed, as noise dominates the channel characteristics. As the noise level drops to 5 to 15 dB, a clear difference emerges. The metamaterial-modulated channel shows a 50% to 200% higher BER than the ideal modulation channel, with the performance gap increasing for more complex modulation schemes. The BPSK, QPSK, and 8-PSK metamateiral modulation requires a 1.5 dB, 2.5 dB, and 4 dB higher SNR to achieve the same level of BER of around $1 \times 10^{-4}$ with the ideal modulation. This is primarily attributed to the increased number of constellation points in higher-order modulation schemes, which amplifies the negative effects of amplitude and phase variations caused by the metamaterial. These variations increase the likelihood of misjudgments during decoding. As the noise ratio declines to 20 to 25 dB, the noise margin becomes negligible compared to the constellation spacing. Under these conditions, the BER for all modulation schemes drops to minimal levels, and the performance of the metamaterial-modulated channels becomes comparable to that of ideal modulation.

Figures 9(b), (c) and (d) present the received constellation diagram of the metamaterial BPSK, QPSK and 8-PSK modulation under an $E_b/N_0$ value of 20 dB. The green dots represent the received sample points from the noisy channel, while the red crosses indicate the ideal constellation positions for each modulation scheme. It is evident that most received constellation points deviate from their ideal reference positions, a result of both channel noise and phase or amplitude fluctuations introduced by the metamaterial. As the number of constellation points increases within higher-order modulation schemes, the constellation points are more densely packed. This will result in the constellation shift caused by metamaterial modulation showing a stronger effect on the noise margin suppression, making the system increasingly sensitive to noise during modulation. This matches the result from the BER analysis.

In addition to AWGN, phase noise is another critical factor influencing the performance of phase modulation schemes, typically arising from thermal noise and local oscillator defects. Phase noise can induce rotation and dispersion of constellation points around the origin, thereby degrading modulation accuracy and the noise margin. Figures 9(e) and 9(f) present the simulated BER versus SNR performance for QPSK and 8-PSK modulation under varying phase noise levels ranging from –85 dBc/Hz to –98 dBc/Hz. For both modulation types, BER performance begins to diverge noticeably when $E_b/N_0$ exceeds 20 dB and approaches a constant value where phase noise becomes the dominant degradation factor. In comparison, the metamaterial-based modulation exhibits a notably higher BER, primarily due to its inherent phase errors, which compromise the system’s robustness against random phase fluctuations. When both AWGN and phase noise are considered simultaneously, the metamaterial-based modulation requires approximately 6.5 dB higher SNR to achieve the same BER level as the ideal modulation. Figure 9(g) further presents the BER performance considering only phase noise. As expected, 8-PSK modulation exhibits weaker phase noise immunity than QPSK, owing to the closer spacing of its constellation points. In a direct comparison between the two modulation methods, the metamaterial-based system would require approximately 1 dBc/Hz improvement in phase noise for QPSK and 3 dBc/Hz for 8-PSK to match the BER performance of the ideal modulation scheme.

Overall, the BER analysis highlights the potential of metamaterial modulation for wireless communication while also identifying key limitations. The observed performance gap in moderate noise conditions highlights the importance of mitigating amplitude and phase variations introduced by the metamaterial to enhance its robustness, particularly for higher-order modulation schemes.

### Challenges and discussion

In conventional on-chip wireless transmission systems, directional signal delivery and target selection are typically achieved through the beam-forming capabilities of on-chip antennas. Works^49,50^ demonstrates the use of phased-array antennas to enable beamforming and selective signal transmission across intra- and inter-chip channels. The given phase array employed a Butler matrix to achieve quadrature phase shifting, and a $2 \times 2$ circular patch planar array facilitates directional beam steering toward the four diagonal directions. However, such phased-array implementations still incur substantial area overhead and exhibit noticeable sidelobes (approximately 1.3 dBi), which can lead to interference and power inefficiency.

The digital coding metamaterial proposed in this work offers an alternative approach for achieving directional signal delivery without relying on the intrinsic radiation pattern of the antenna. Using a near-omnidirectional antenna with four modulation metamaterials, the system can achieve directional transmission toward the four diagonal directions through post-radiation modulation. Additionally, the post-radiation modulation exhibits a distinct advantage for simultaneous multi-target data transmission from a single source antenna. Independent bitstreams can be applied to different metamaterial units for parallel modulation, enabling concurrent communication with multiple receivers. In contrast, the phased-array-based approach inherently supports transmission to a single target at a time, as the signal is encoded before radiation. Target switching in such systems requires completing the current transmission cycle (often governed by a token-based protocol), followed by reconfiguring the Butler matrix and loading a new bitstream for the next transmission. The elimination of this sequential switching process in the proposed metamaterial modulation architecture could significantly enhance system responsiveness, making it particularly suitable for delay-sensitive intra-chip applications.

Nevertheless, the metamaterial-based modulation system still exhibits relatively obvious limitations at the current stage of research. As revealed by the BER analysis, the proposed modulation metamaterial suffers from higher insertion loss and reduced noise immunity compared to conventional circuit-based modulators, which arise from factors such as the coupled amplitude–phase response in the resonance unit cells, inter-column coupling, and parasitic components introduced during fabrication. Consequently, it requires an additional 8–10 dB of signal amplification to compensate for the insertion loss and maintain acceptable noise performance. This may diminish the energy and throughput advantages of metamaterials modulation in multi-target transmission.

Several procedures may be applied to improve the noise performance of the metamaterial modulation. First, the current metamaterial supports only binary coding sequences for QPSK and 8-PSK modulation. Adopting Grey-coding could significantly reduce the bit-error rate (BER) by minimising the impact of single-bit errors on decoding accuracy. However, this would require either a redesign of the switching states in the metamaterial unit cells or the integration of an additional code converter into the metamaterial driving circuit. Secondly, the current phase-shifting unit cells are tuned using PIN diodes, which support only two discrete states: ’0’ or ’1’. Instead, implementing varactor capacitance diodes could provide finer control over both phase and amplitude components by adjusting the input voltage level^30,31^, thereby achieving more accurate constellation positions and reducing the BER. Third, support for multiple modulation schemes enables the system to adapt dynamically and flexibly to varying noise conditions. By selecting the optimal modulation scheme based on external noise levels, the system can achieve a better balance between data rate and BER.

In addition to BER performance, the proposed metamaterial structure incurs a notable area overhead (5.66 $mm^2$), which remains significantly larger than that of a conventional modulation-circuit implementation. A multi-target transmission configuration further amplifies this overhead by requiring multiple sets of metamaterial modulators. It is important to note that mainstream RF CMOS technology nodes (e.g., 65 nm and 28 nm) provide the patterning precision and multiple metal interconnect layers that far exceed the implementation requirements of on-chip antennas and the proposed modulation metamaterial. Implementing the metamaterial within these CMOS circuit platforms is neither economically efficient nor technologically necessary. A more suitable solution is to fabricate the modulation metamaterial using a mature SOI silicon photonics platform^51^, which offers both the required device layers and substantially lower fabrication costs. The SOI PIN-based tunable structure in the proposed metamateiral is highly compatible with standard carrier-injection silicon optical modulator processes, sharing similar P+/N+ doping regions, PN junction contact vias, and aluminium contact layers. Importantly, the SOI photonics platform offers a significantly lower cost per unit area than CMOS RF circuit processes. As a result, although the metamaterial does not offer an area advantage relative to a conventional IQ-mixer-based modulation circuit, its overall implementation cost can be substantially lower. This advantage makes the proposed metamaterial architecture a more economically viable and accessible option.

## Conclusion

We demonstrated a transmissive digital-coding metamaterial with hybrid digital modulation and beam-steering capabilities designed for intra-chip millimeter-wave interconnects. The proposed metamaterial is constructed with three columns of phase-shifting unit cells, capable of achieving phase shifts of $180^{\circ }, 90^{\circ },\,\hbox {and}\, 45^{\circ }$ on a 70 GHz carrier wave through ’0’ to ’1’ state switching. The digital-coding metamaterial supports two primary operation modes. In modulation mode, it enables multiple phase-shifting modulation schemes, including BPSK, QPSK, and 8-PSK. In modulation and beam-steering mode, it facilitates BPSK modulation combined with dynamic beam steering over a range of $\pm 28^{\circ }$. Leveraging the capabilities of the proposed digital-coding metamaterial, we also introduce a novel on-chip wireless transmission architecture, which replaces conventional signal modulators and mixer circuits with the coding metamaterial. When multiple sets of metamaterials operate in conjunction with a broadcasting transmitter (TX) antenna, data can be encoded and simultaneously transmitted to multiple spatially separated receiver targets. This approach is expected to significantly increase the throughput of a single on-chip antenna while minimising interference and target-switching delays across transmission channels. At this stage, several challenges remain in implementing the coding metamaterial. One notable issue is the relatively weak noise immunity, which arises from coding inaccuracies. Additionally, the cascaded structure contributes to relatively high insertion losses ($\sim$ 4 dB) due to the cumulative losses incurred as EM waves propagate through each column of unit cells. Future research could address these limitations by exploring the use of multi-bit modulation unit cells or varactor diodes as tunable components, which may help reduce insertion losses and the overall footprint of the coding metamaterial.

## Data Availability

The datasets used and/or analysed during the current study are available from the corresponding author on reasonable request.
